# Purification of Boron Nitride Nanotubes Enhances Biological Application Properties

**DOI:** 10.3390/ijms21041529

**Published:** 2020-02-24

**Authors:** Soul-Hee Lee, Myung Jong Kim, Seokhoon Ahn, Byumseok Koh

**Affiliations:** 1 Institute of Advanced Composite Materials, Korea Institute of Science and Technology, 92 Chudong-ro, Bongdong-eup, Wanju-gun 55324, Jeollabuk-do, Korea; happy-snow@kist.re.kr (S.-H.L.); myung@kist.re.kr (M.J.K.); 2 Department of Bioactive Material Sciences, Chonbuk National University, 567 Baekje-daero, Jeonju 54896, Jeollabuk-do, Korea; 3 Department of Nanochemistry, Gachon University, Sungnam 13120, Korea; 4 Therapeutics and Biotechnology Division, Korea Research Institute of Chemical Technology, 141 Gajeong-ro, Yuseong-gu, Daejeon 34114, Korea

**Keywords:** boron nitride nanotube, purification, encapsulation, dispersion

## Abstract

Commercially available boron nitride nanotubes (BNNTs) and their purified form (pBNNTs) were dispersed in aqueous solutions with various dispersants, and their cytotoxicity and drug encapsulation capacity were monitored. Our data suggest that pBNNTs showed an average increase in dispersibility of 37.3% in aqueous solution in the presence of 10 different dispersants. In addition, 100 μg of pBNNTs induced an average decrease in cytotoxicity of 27.4% compared to same amount of BNNTs in normal cell lines. The same amount of pBNNTs can encapsulate 10.4-fold more drug (camptothecin) compared to BNNTs. These data suggest that the purification of BNNTs improves several of their properties, which can be applied to biological experiments and are thus essential in the biological application of BNNTs.

## 1. Introduction

Boron nitride nanotubes (BNNTs) consist of boron and nitrogen atoms arranged in a hexagonal lattice [1,2,3,4,5]. Due to their structural similarity, both BNNTs and carbon nanotubes (CNTs) have special characteristics, including a high thermal conductivity and mechanical strength [6,7,8,9]. BNNTs can be used for aerospace applications when boron and light isotope of boron integration to structural materials improves their radiation-shielding properties and strength. It is also intensively studied for ultraviolet (UV) shielding applications [10,11,12,13]. 

CNTs and BNNTs were extensively explored for biological purposes, including their potential application to hydrophobic drug carrier carriers [14,15,16]. Due to their available hydrophobic inner space, ability to disperse in aqueous solution in the presence or absence or dispersants, and large aspect ratio, both CNTs and BNNTs can either be endocytosed or directly penetrate cell membranes, allowing targeted delivery of hydrophobic drugs. The degree of BNNTs application in gene delivery/cell imaging and stimulation were also examined by researchers [17]. However, because of their their less efficient dispersion in aqueous solution and cytotoxicity, BNNTs are not as widely applied as CNTs for biological experiments.

Current commercially available BNNTs have low (~50%) purity and contain significant amounts of hexagonal boron nitride (h-BN) and BNH compounds such as ammonia borane [18,19,20]. The low level of purity and the impurities contained in BNNTs can contribute to BNNT-induced cytotoxicity as well as hinder their dispersion in aqueous solution [21,22,23]. Additionally, this low purity of BNNT can hinder its application as a potential drug carrier, like CNTs due to poor encapsulation capacity. All these aspects of low purity BNNTs greatly hinder their potential usage in biological application. Currently, many careful BNNT purification methods have been developed [24,25,26,27,28,29] in order to produce highly purified BNNTs; however, side-by-side comparison studies between unpurified BNNTs and purified BNNTs (pBNNTs) in biological applications are rare. 

In order to examine the effectiveness of pBNNTs in biological applications, and to fill the research gap and determine the effect of BNNT purification on their biological properties, including cytotoxicity, water dispersibility, and drug encapsulation, we conducted systematic studies comparing BNNTs and pBNNTs. 

## 2. Results

**Figure 1:**
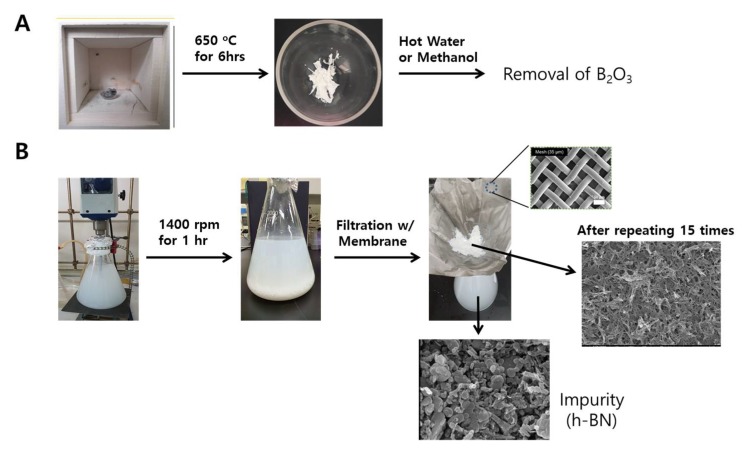
Boron nitride nanotubes (BNNT) purification processes used in the present case: (**A**) process to remove boron element and (**B**) process to remove hexagonal boron nitride (h-BN) impurities.

Purification and characterization of BNNTs. The commercial BNNT sample consists of <50% BNNTs, ~25% B, and ~25% h-BN and BNH derivatives. To remove the B element, BNNT samples were heated at 650 °C for 6 hours in air to oxidize amorphous boron. BNNT purification processes that we conducted are shown in Figure 1. 

**Figure 2:**
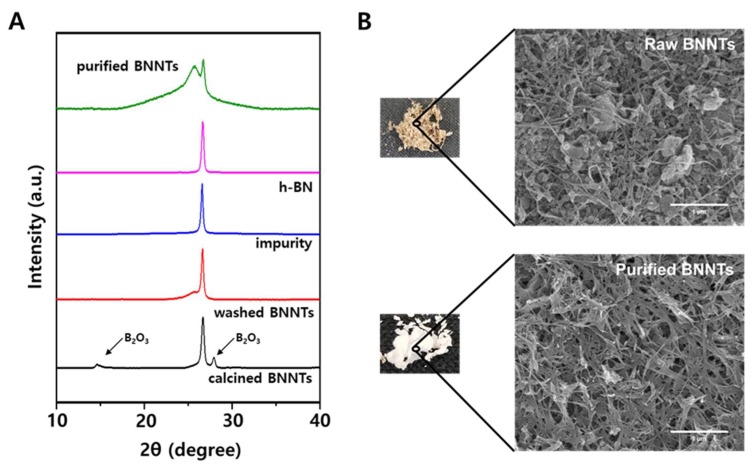
(**A**) X-ray diffraction spectrum and (**B**) SEM images of before and after the purification of boron nitride nanotubes.

Since amorphous boron is oxidized to B_2_O_3_ and boric acid during this process, the sample color changed from gray to white, as shown in Figure 2B [18]. These impurities could be removed by washing the sample with hot water or methanol. The remained impurities are mainly h-BN particles with various sizes, which are difficult to separate from BNNTs because of their similar chemical properties. Recently, it was reported that h-BN impurities could be selectively removed by ultrasonically assisted diffusion separation [18,30]. The BNNTs and h-BN particles were dispersed in water by ultrasonication for 10 min, and the solution was vigorously stirred for 1 hr at 1400 rpm and filtered using a porous membrane (Stainless Steel Woven Wire Mesh) with 35 micrometer pore size. During this process, h-BN particles, which have a lower aspect ratio and smaller size than BNNTs, diffuse through the membrane, whereas BNNTs are collected on the membrane due to their higher aspect ratio. This process was repeated over 15 times to obtain the purified BNNT. This purification process was characterized by using XRD and SEM as shown in Figure 2. The calcined BNNTs clearly show the B_2_O_3_ peaks at 15° and 28° in XRD. After washing process using hot water or methanol, these peaks were clearly disappeared, supporting the removal of B_2_O_3_ impurities. The separation of h-BN and BNNTs was conducted by repeating filtration process using porous membrane. During the filtration, BNNT-rich samples were collected on the membrane where h-BN impurities were diffused through the membrane as shown in SEM images in Figure 1. This process is more clearly monitored using X-ray diffraction (XRD) as shown in Figure 2A. The washed BNNTs showed two different peaks where the broad peak at 25.8° and the sharper peak at 26.7° is originated by BNNTs and h-BN impurities, respectively. During the filtration process, the impurities were collected and characterized by using SEM and XRD. The XRD peak of the collected impurity is matched with that of commercial h-BN. This result and the SEM image of the collected impurity shown in Figure 1 reveal that h-BN impurity could be separated from BNNTs by using this method. However, although this process was repeated until the filtrated solution was clear, the purified BNNTs sample still contained h-BN impurity, as shown in Figure 2a. The quantification of remained h-BN content was conducted by using IR analysis [18]. The ratio of out-of-plane buckling mode (R) and in-plane optical mode, transverse optical (TO) mode of purified BNNTs was 0.208, corresponding to 22% h-BN content (Figure S1). This value is similar to that obtained from purified BNNT using similar membrane filtration in reference 17. The SEM images before and after purification in Figure 2 clearly shows that a BNNT-rich sample can be obtained through these processes. 

### 2.1. Dispersion of BNNTs in Aqueous Solutions

**Figure 3:**
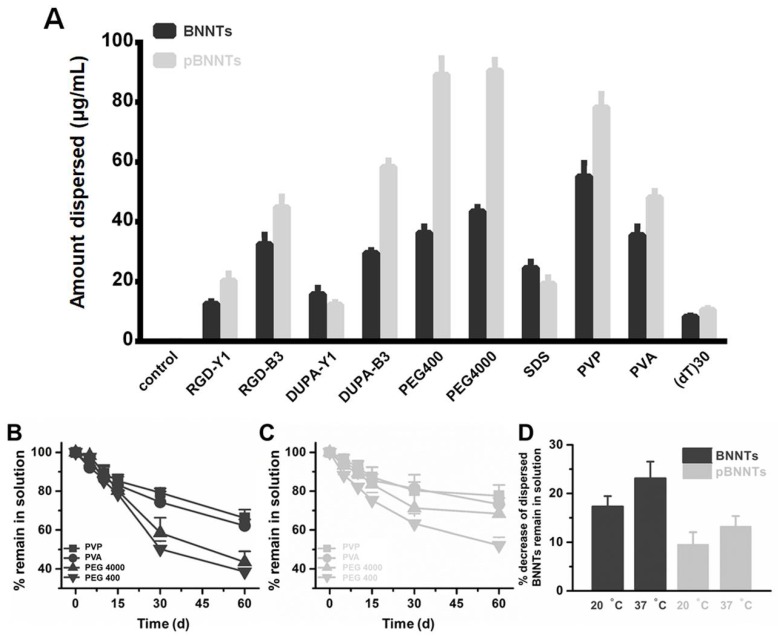
(**A**) The dispersions of BNNTs and their purified form (pBNNTs) in aqueous solution with dispersants. Dispersion stabilities of (**B**) BNNTs and (**C**) pBNNTs with dispersants. (**D**) % decrease of dispersed BNNTs remain in solution at 20 and 37 °C. Error bars represent the standard deviation of three replicates.

The dispersion of BNNTs before and after purification in the presence of various dispersants was monitored. We have selected list of dispersants that were extensively studied to disperse either CNTs or BNNTs in previous studies by other groups. Data suggest that pBNNTs showed an increased amount of dispersion in the presence of the dispersants used (29.2%, 13.5%, 86.3%, 121.6%, 109.3%, 19.2%, 13.5%, and 3.5% increases for Arg-Gly-Asp (RGD), pyrazine, RGD-pyrazine, polyethylene glycol 400 (PEG400), polyethylene glycol 4000 (PEG4000), polyvinylpyrroidone (PVP), polyvinyl alcohol (PVA), and DNA oligo {TTT TTT TTT TTT TTT TTT TTT TTT TTT TTT, (dT)_30_}, respectively, while pBNNTs showed no increase in the amount of dispersion in the presence of fluorescein isothiocyanate (FITC) and sodium dodecyl sulfate (SDS) (11.1 and 12.4% decreases, respectively) compared to BNNTs (Figure 3A). We further tested the stabilities of BNNTs and pBNNTs dispersed in aqueous solutions in the presence of PVP, PVA, PEG 400, and PEG 4000. Data suggest that pBNNTs dispersed in water showed an increase in the amount left in solution (7.6%, 2.9%, 83.6%, and 43.9% increases for PVP, PVA, PEG 4000, and PEG 400, respectively, after 60 d of storage), compared to unpurified BNNTs (Figure 3B,C). We also monitored % decrease of dispersed BNNTs that remained in solution at 20 and 37 °C, and data suggested that dispersed pBNNTs are stable at both conditions, compared to unpurified BNNTs (Figure 3D).

### 2.2. Cytotoxicity Induced by Dispersed and Raw BNNT and pBNNTs

**Figure 4:**
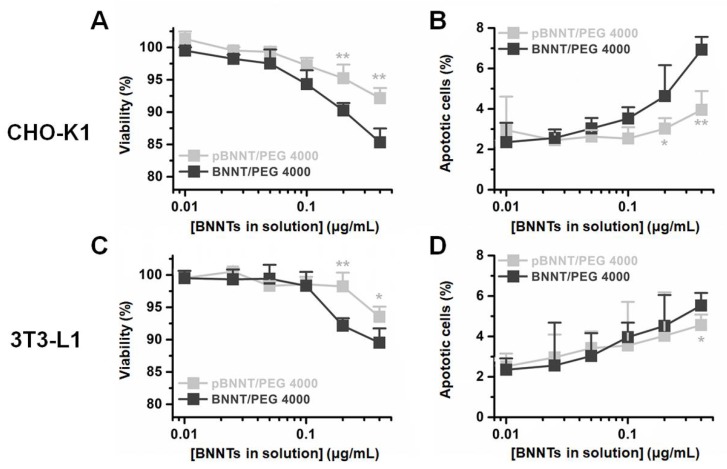
Water dispersed BNNT/PEG 4000- and pBNNT/PEG 4000-induced cytotoxicity in (**A**) CHO-K1 and (**C**) 3T3-L1 cells. Percentages of apoptotic (**B**) CHO-K1 and (**D**) 3T3-L1 cells after incubation with BNNTs and pBNNTs. Error bars represent the standard deviation of tee replicates. * for *p* < 0.05, ** for *p* < 0.01.

**Figure 5:**
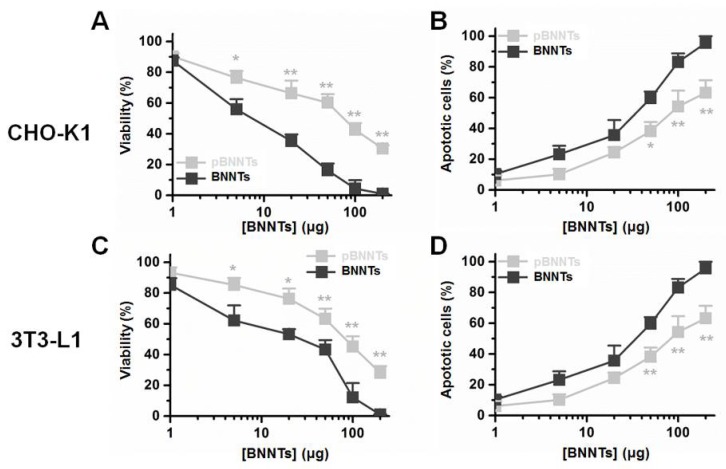
BNNT- and pBNNT-induced cytotoxicity in (**A**) CHO-K1 and (**C**) 3T3-L1 cells. Percentages of apoptotic (**B**) CHO-K1 and (**D**) 3T3-L1 cells after incubation with BNNTs and pBNNTs. Error bars represent the standard deviation of tee replicates. * for *p* < 0.05, ** for *p* < 0.01.

We tested the cytotoxicity induced by dispersed and undispersed BNNTs and pBNNTs with two non cancer cell lines, CHO-K1 and 3T3-L1. BNNTs and pBNNTs dispersed with PEG 4000 in aqueous solution were used for test cytotoxicity. pBNNT/PEG 4000 showed 8.9% and 5.6% less cytotoxicity in CHO-K1 and 3T3-L1 cells, respectively, while inducing 3.4% and 0.8% less percentages of CHO-K1 and 3T3-L1 cells undergoing apoptosis, respectively, compared to BNNT/PEG 4000 (Figure 4). For nondispersed BNNTs and pBNNTs, one hundred micrograms of pBNNTs induced 3.72 and 9.79-fold lower cytotoxicities for CHO-K1 and 3T3-L1, compared to same amount of BNNTs (Figure 5A,C). One hundred micrograms of pBNNTs induced 25.9% and 28.9% decreases in the CHO-K1 and 3T3-L1 cells undergoing apoptosis, compared to BNNTs (Figure 5B,D). 

### 2.3. Drug Encapsulation and Drug Delivery with BNNTs and pBNNTs

**Figure 6:**
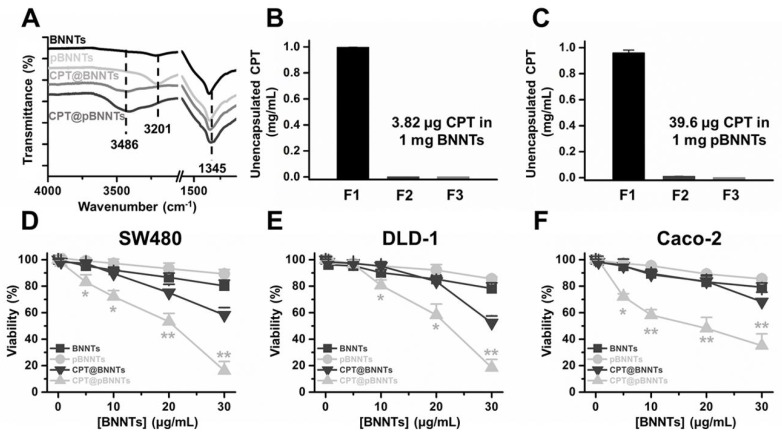
(**A**) Raman spectra of BNNTs and pBNNTs after CPT encapsulation. Estimation of the amount of CPT encapsulated in (**B**) BNNTs and (**C**) pBNNTs. Cytotoxicity induced by control BNNTs, pBNNTs, and CPT, encapsulated by BNNTs and pBNNTs in (**D**) SW480, (**E**) DLD-1, and (**F**) Caco-2 colorectal cancer cells. Error bars represent the standard deviation of tee replicates. * for *p* < 0.05, ** for *p* < 0.01.

The camptothecin (CPT) molecule was used for encapsulation in BNNTs. Raman spectra show a more prominent peak from CPT (3486 cm^−1^) in pBNNTs compared to in BNNTs (Figure 6A). The estimated amount of encapsulated CPT suggests that pBNNTs can encapsulate 10.37-fold more CPT compared to unpurified BNNTs (Figure 6B,C). A concentration of 30 μg/mL CPT encapsulated by pBNNTs was 42.0%, 33.8%, and 33.1% more toxic in SW480, DLD-1, and Caco-2 colon cancer cells, respectively, compared to CPT encapsulated by BNNTs (Figure 6D–F). Both blank BNNTs and pBNNTs induced >18% decrease in SW480, DLD-1, and Caco-2 viabilities.

## 3. Discussion

Comparison studies on biological applications with BNNTs and pBNNTs were conducted. We first characterized the BNNTs before and after purification. We then conducted comparative dispersion studies of BNNTs in aqueous solution. Common dispersants (PEG 400, PEG 4000, and ssDNA) as well as fluorophores [31,32,33] and BNNT dispersants (PVA and PVP) were used to disperse BNNTs and pBNNTs in aqueous solution. In the same experimental conditions, pBNNTs showed an increased amount of dispersion in aqueous solution in the presence of 8 out of the 10 dispersants that were used. BNNTs showed more dispersion than pBNNTs in the presence of FITC and SDS; however, the differences were >13% for both dispersants. These data suggest that the purification of BNNTs helps BNNTs to be better dispersed in aqueous solution. We also tested whether these dispersed BNNTs and pBNNTs can retain their dispersibility over the time course of the storage period. We and several other groups previously reported that CNTs dispersed in aqueous solution can be stable for up to several months [34,35,36,37]. However, unlike pBNNTs, dispersed BNNTs quickly aggregated and sedimented over the 60 d storage period. We monitored the amounts of dispersed BNNTs and pBNNTs remaining in solution with four dispersants (PVP, PVA, PEG 4000, and PEG 400), and on average, 52.6% of dispersed BNNTs still remained in solution after 60 day of storage, while 67.8% of pBNNTs still remained in solution. Purification of BNNTs both increases the dispersibility as well as stability in aqueous solution in the presence of dispersants. Next, we monitored the cytotoxic effects of BNNTs and pBNNTs on normal cell lines. We have tested both water dispersed (aided by PEG 4000) and nondispersed BNNTs and pBNNTs powder for monitor noncancer cell cytotoxicity. Various amounts (1–200 μg) of BNNTs and pBNNTs were applied to CHO-K1 and 3T3-L1 cells, and the cell viability as well as percentage of cells undergoing apoptosis was determined. One hundred micrograms of pBNNTs induced 54.8% and 57.4% decreases in the viability of CHO-K1 and 3T3-L1 cells, respectively, compared to the control, while the same amount of BNNTs induced 87.9% and 95.7% decreases in viability, respectively (Figure 5A and C). The percentage of CHO-K1 and 3T3-L1 cells undergoing apoptosis showed similar results. CHO-K1 and 3T3-L1 cells incubated with 100 μg of pBNNTs showed 59.6% and 54.2% undergoing apoptosis, while cells incubated with the same amount of BNNTs showed 85.5% and 83.2% undergoing apoptosis. Both cell viability and apoptosis data indicate that the purification of BNNTs in dispersed or nondispersed form reduces their cytotoxicity compared to nonpurified BNNTs. Next, we determined the hydrophobic drug encapsulation ability, as both CNTs and BNNTs have potential applications as drug carriers [38,39]. Hydrophobic anticancer small molecule CPT was used to test encapsulation efficiency of BNNTs and pBNNTs. The topoisomerase I inhibitor CPT can potentially be used to treat colon and small lung cancer. CPT shows potent anticancer efficacy and is often considered one of the most effective anticancer agents. Its cytotoxic effect is mostly caused by blocking the action of topoisomerase I, which is required for relaxing DNA supercoiling process during the cell replication cycle. However, the water insolubility and poor pharmacological profile of CPT significantly hinder its biological applications. One approach is to encapsulate CPT in nanomaterials which can function as a watercompatible outer shell. Because of its needle-like shape and high aspect ratio, BNNTs can either be endocytosis or direct penetrate through cell membranes. In addition, previous studies have suggested that BNNTs can trap hydrophobic molecules in their inner space via hydrophobic−hydrophobic interactions [14]. Our data suggest that purification of BNNTs also increased the estimated amount of encapsulated CPT, as 1 mg of pBNNTs encapsulates 39.6 μg of CPT, while the same amount of BNNTs encapsulated 3.82 μg (Figure 6B,C). Both BNNTs- and pBNNTs-encapsulating drugs were dispersed in aqueous solution with PVP and incubated with three colorectal cell lines. A drug concentration of 30 μg/mL encapsulated by pBNNTs induced 83.8%, 81.5%, and 64.9% decreases in the viability of SW480, DLD-1, and Caco-2 cells, respectively, while the same concentration of drug encapsulated by BNNTs induced 41.8%, 47.7%, and 31.8% decreases in the cell viability (Figure 5D–F). These data can be explained by the amount of CPT encapsulated, as the same concentration of pBNNTs enables the encapsulation of more CPT compared to BNNTs. 

Although pBNNTs showed better properties for biological applications than unpurified BNNTs, there is still room for improvement. The purity of pBNNTs is about 75 %, and this does not match with the high purity of commercially available purified CNTs (> 98%). The same amount of purified functionalized CNTs induces only 36.2% cytotoxicity in the cells we have tested (data not shown). In addition, functionalized or unfunctionalized CNTs with dispersants can still be dispersed in aqueous solution more than the pBNNTs tested. The hydrophobic drug encapsulation capacity (100.9 μg for 1 mg of functionalized CNTs [40]) of CNTs is > 2.5-fold greater than that of pBNNTs. Further purification technologies can improve these properties for better biological applications.

In summary, we have demonstrated a side-by-side comparison between pBNNTs and BNNTs in terms of their properties that can be applied for biological purposes. Purification of BNNTs improves their dispersibility in aqueous solution, lowers their cytotoxicity, and allows them to have a greater capacity of carrying hydrophobic drugs than unpurified BNNTs. These purified BNNTs can be applied for various biological experiments, including drug delivery.

## 4. Experimental Section

### 4.1. Materials

Boron nitride nanotubes (BNNTs, avg. diam. 5 nm, nanotube, surface area >100 m^2^/g) were purchased from Sigma-Aldrich (St. Louis, MO, USA). 3T3-L1, Caco-2, SW480, and DLD-1 cells were purchased from the American Tissue Type Collection (ATCC, Manassas, VA, USA). CHO-K1 was obtained from the Korean Cell Line Bank (KCLB, Seoul, Korea). Dulbecco’s Modified Eagle Medium (DMEM), Ham’s F-12 (F-12), fetal bovine serum (FBS), Dulbecco’s phosphate-buffered saline (DPBS), and penicillin streptomycin were purchased from Thermo Fisher Scientific (Waltham, MA, USA). RGD, pyrazine, FITC, PEG 400 and 4000, SDS, PVA (mol wt. 40,000), and PVA (mol wt. 47,000) were purchased from Sigma-Aldrich. Short single-stranded DNA, (dT)_30_, was purchased from Integrated DNA Technologies (Coralville, IA, USA). Molecular-biology-grade pure water was purchased from Biosesang (Seongnam, Korea). The cell counting kit-8 (CCK-8) was purchased from Dojindo (Kummamoto, Japan), and the apoptosis detection kit was purchased from Merck Millipore (Burlington, MA, USA). Camptothecin (CPT, >90%) was purchased from Sigma-Aldrich.

### 4.2. Purification and Characterization of BNNTs

The 1 g of received BNNT sample was heated at 650 °C for 6 h for the oxidation of amorphous boron. The oxidized sample was dispersed in 2 L of hot methanol and stirred for 1 h to remove the oxidized boron. This process was repeated two times in order to completely remove oxidized boron chemicals. After the amorphous boron was removed, the BNNT sample, which was obtained by filtration, was dispersed in water by ultrasonication for 10 min, and the solution was vigorously stirred for 1 h at 1400 rpm and filtered using a porous membrane (Stainless Steel Woven Wire Mesh) with 35 micrometer pore size. This process was repeated over 15 times until the filtered solution became clear to obtain a BNNT-rich sample (Figure S2). 

### 4.3. Dispersion of BNNTs and pBNNTs in Aqueous Solution

One milliliter of 1 mg/mL RGD, pyrazine, RGD-pyrazine, SDS, PVA, PVP, PEG 400, PEG 4000, and (dT)_30_, and 0.05 mg/mL FITC in molecular-biology-grade water, were added to 1 mg of BNNT and pBNNTs, respectively. The BNNTs in aqueous solution were than sonicated for 1 h in an ultrasonic bath (Branson Ultrasonics Corporation, St. Louis, MO, USA). Ice was added every 10 minutes to prevent overheating. Sonicated samples were then centrifuged at 17,900 g for 1 h (Eppendorf 5427R, Hamburg, Germany), and the supernatants were collected. The amount of BNNTs dispersed in aqueous solution was estimated by absorbance measurements at 600 nm (UV-1800, Shimadzu Corporation, Kyoto, Japan). After the designated storage day, BNNTs in aqueous solution were re-centrifuged (17,900 g for 1 h) and the absorbance at 600 nm was measured.

### 4.4. Cytotoxicity of BNNTs and pBNNTs

CHO-K1 cells were cultured in DMEM/F-12 media supplemented with 10% FBS and 1% penicillin-streptomycin. 3T3-L1 cells were maintained in DMEM supplemented with 10% FBS and 1% penicillin-streptomycin. One hundred microliters of 1 × 10^4^ CHO-K1 and 3T3-L1 cells was seeded on a 96 well cell culture plate. Samples of dispersed 0.01, 0.025, 0.05, 0.1, 0.2, and 0.4 μg/mL BNNTs and pBNNTs in aqueous solution with PEG 4000 and undispersed 1, 5, 20, 50, 100, and 200 μg of BNNTs and pBNNTs were added to the cells and incubated for 48 h. After 48 h, the cell viability was measured with a CCK-8. The apoptosis rate of cells after BNNT exposure was determined with an apoptosis detection kit and Muse^®^ cell analyzer (Merck Millipore). 

### 4.5. Drug Encapsulation in BNNTs and Drug Delivery

The encapsulation of camptothecin in BNNTs was achieved by simple incubation with mild agitation, as described previously [31]. One milligram of CPT was solubilized in 1 mL of DMSO and incubated with 1 mg of BNNTs and pBNNTs. After 5 d of stirring, the BNNT samples were centrifuged at 17,900 g for 1 h and the supernatant (F1) was collected. The surface of BNNTs was carefully washed twice and washed fractions of camptothecin residuals (F2 and F3) were collected (with centrifugation at 17,900 g for 1 h). Amounts of camptothecin in each fraction of solution were carefully measured with LC/MS and compared with amount of camptothecin in original camptothecin solution (before BNNT incubation). After the final washing step, the samples were dried to yield CPT@BNNTs and CPT@pBNNTs. CPT@BNNTs and CPT@pBNNTs were then dispersed in molecular-biology-grade water in the presence of 1 mg/mL PVP. No detectable amount of camptothecin in CPT@BNNTs dispersed in water was observed, indicating no unencapsulated camptothecin is available in the solution. For the delivery of CPT@BNNTs, 100 μL of 1 × 10^4^ SW480, Caco-2, and DLD-1 cells was seeded on 96 well cell culture plates. After 24 h, 10 μL of the designated amount of CPT@BNNTs was added to the cells, resulting in final BNNT concentrations of 5, 10, 20, and 30 μg/mL. After 48 h of incubation, the cells were washed with DPBS and the cell viability/apoptotic rate were measured. 

### 4.6. Statistical Analyses

Statistical analysis was performed using GraphPad Prism (version 6, GraphPad Software, Inc., San Diego, CA, USA) and Origin 8.5 (OriginLab Corporation, Northampton, MA, USA). Each experiment was performed in triplicate, and values are expressed as the mean ± standard deviation (SD). Statistical significance is denoted as * for *p* < 0.05 and ** for *p* < 0.01.

## Supplementary Materials

Supplementary materials can be found at https://www.mdpi.com/1422-0067/21/4/1529/s1.
