# Supplementary material for: Purification of Boron Nitride Nanotubes Enhances Biological Application Properties
Source: Int J Mol Sci. 2020 Feb 24;21(4):1529. doi: 10.3390/ijms21041529 (PMC7073224; doi:10.3390/ijms21041529)
Supplement: Supplementary file 1 [file ijms-21-01529-s001.pdf]

Purification Processes

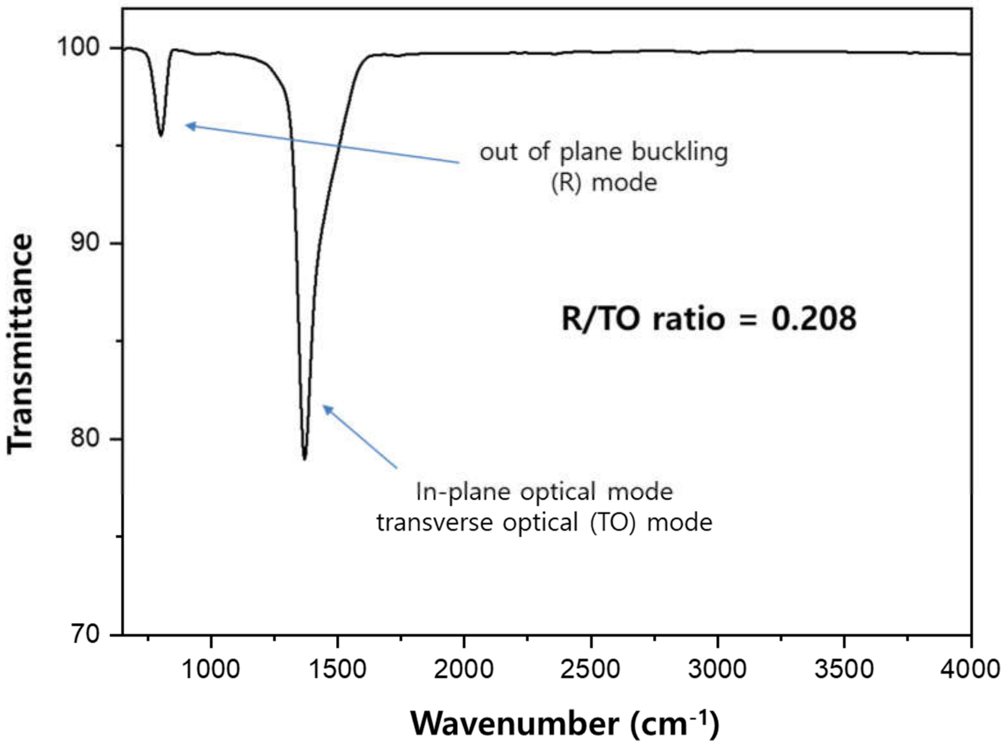

Figure S1. IR spectrum of purified BNNT. The R/TO ratio of 0.208 is corresponding to 22% h-BN content and 78% BNNT based on IR analysis reported in *Nanoscale Advanced*, 2019, 1, 1693-2701.

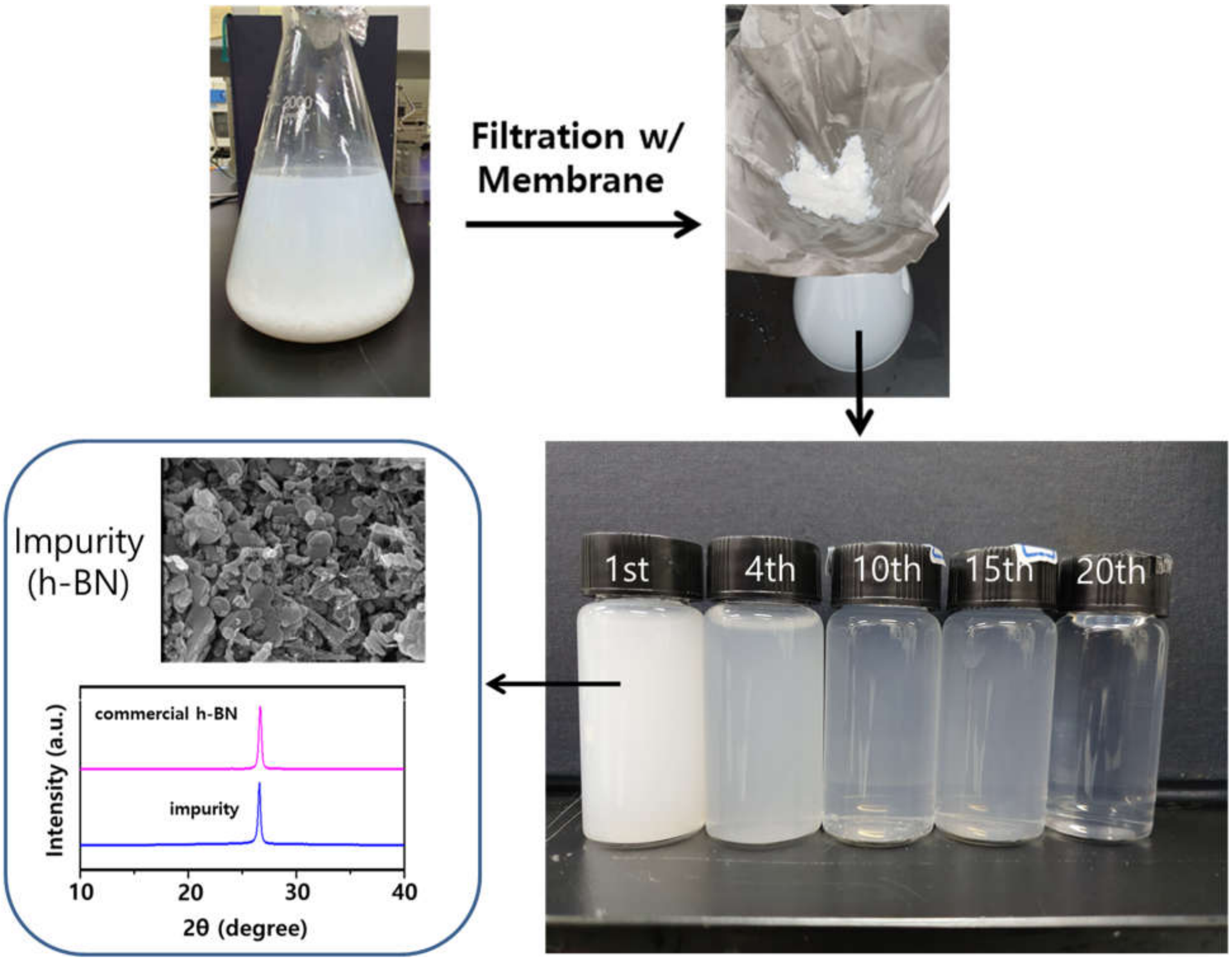

Figure S2. The filtered solution during repeating the filtration process using porous membrane.
